# TWEAK/Fn14 Activation Participates in Skin Inflammation

**DOI:** 10.1155/2017/6746870

**Published:** 2017-09-06

**Authors:** Qilu Liu, Shengxiang Xiao, Yumin Xia

**Affiliations:** Department of Dermatology, The Second Affiliated Hospital, School of Medicine, Xi'an Jiaotong University, Xi'an, China

## Abstract

Tumor necrosis factor- (TNF-) like weak inducer of apoptosis (TWEAK) participates in multiple biological activities via binding to its sole receptor—fibroblast growth factor-inducible 14 (Fn14). The TWEAK/Fn14 signaling pathway is activated in skin inflammation and modulates the inflammatory responses of keratinocytes by activating nuclear factor-*κ*B signals and enhancing the production of several cytokines, including interleukins, monocyte chemotactic protein-1, RANTES (regulated on activation, normal T cell expressed and secreted), and interferon gamma-induced protein 10. Mild or transient TWEAK/Fn14 activation contributes to tissular repair and regeneration while excessive or persistent TWEAK/Fn14 signals may lead to severe inflammatory infiltration and tissue damage. TWEAK also regulates cell fate of keratinocytes, involving the function of Fn14-TNF receptor-associated factor-TNF receptor axis. By recruiting inflammatory cells, promoting cytokine production, and regulating cell fate, TWEAK/Fn14 activation plays a pivotal role in the pathogenesis of various skin disorders, such as psoriasis, atopic dermatitis, cutaneous vasculitis, human papillomavirus infection and related skin tumors, and cutaneous autoimmune diseases. Therefore, the TWEAK/Fn14 pathway may be a potential target for the development of novel therapeutics for skin inflammatory diseases.

## 1. Introduction

Tumor necrosis factor- (TNF-) like weak inducer of apoptosis (TWEAK) is a member of the TNF ligand superfamily and is initially described as an inducer of apoptosis in transformed cell lines [1]. TWEAK acts via binding to its sole receptor—fibroblast growth factor-inducible 14 (Fn14), the smallest member of the TNF receptor (TNFR) superfamily [2]. The specificity of TWEAK binding to Fn14 has been confirmed in multiple experiments [3]. TWEAK is broadly expressed by monocytes, dendritic cells, and natural killer (NK) cells, and macrophages/monocytes are the main source of soluble TWEAK (sTWEAK) in inflammatory tissues [4–8]. The immune organs, including the spleen, lymph nodes, and appendix, also express TWEAK [1, 4]. TWEAK has also been detected in various tumor cell lines [9–12]. Fn14 is widely expressed in various tissues including the skin, heart, brain, kidney, colon, small intestine, skeletal muscle, and pancreas [2, 13–17]. In normal tissues, the expression of TWEAK and Fn14 is relatively low. Elevated expression of TWEAK and Fn14 is usually seen in response to stress, tissue injury, or remodeling [18–20]. Optimal TWEAK-mediated activation of Fn14 promotes productive tissue responses after injury; however, excessive or persistent Fn14 upregulation and TWEAK/Fn14 activation often induce various pathological responses [21]. TWEAK/Fn14 signaling pathway participates in multiple biological activities, including the proliferation, differentiation, migration and death (apoptosis/necrosis) of cells [22–28], angiogenesis [2, 29], and inflammatory responses [2, 30].

Inflammation is one of the basic characteristics of skin disorders, especially the chronic inflammatory diseases that include psoriasis, atopic dermatitis (AD), cutaneous vasculitis, and cutaneous lupus erythematosus [31]. The occurrence of psoriasis varies according to age and geographic region, with the estimates of prevalence in adults ranged from 0.51% to 11.43% and in children from 0% to 1.37% [32]. AD is even more prevalent among people of any age. In developed countries, the incidence of AD varies in 10% to 20%, whereas it is lower but continues to increase in many developing countries [33]. Cutaneous vasculitis refers to a wide spectrum of diseases characterized by primary or secondary blood vessel inflammation and necrosis in skin [34]. Cutaneous lupus erythematosus is a chronic autoimmune disease, with an estimated incidence of 4.2 per 100,000 people [35]. Skin infections and malignancies are also related to local abnormalities in immune and inflammatory responses [36, 37]. These disorders not only affect skin tissue but also develop extracutaneous or even systemic complications as primary inflammation exacerbates continuously. To suppress exacerbated inflammatory injuries is one of the strategies for treating skin disorders. Under skin inflammation, proinflammatory cytokines as well as chemokines are continuously released, recruiting an infiltration of immune cells. Recently, it was reported that TWEAK/Fn14 interaction increases the expression and/or secretion of various molecules involved in local inflammatory responses [38–40]. Moreover, TWEAK promotes the proinflammatory activities of other cytokines such as TNF-*α*, interleukin- (IL-) 1, IL-6, and interferon-*γ* [28, 41], which also participate in the pathogenesis of inflammatory skin diseases [28, 42, 43]. Therefore, these findings suggest a pivotal role of TWEAK/Fn14 pathway in the mechanism of cutaneous inflammation.

In this review, we update recent advances in the function of TWEAK/Fn14 signals in different skin inflammation and also highlight the potential roles of this pathway as therapeutic target in the management of various skin diseases.

## 2. The Structural Basis of TWEAK/Fn14 Interaction

TWEAK is initially synthesized as type II transmembrane proteins of 249 amino acids and can be cleaved by furin into sTWEAK with biologic activities [44]. The C-terminal extracellular domain of TWEAK contains the receptor-binding subdomain, which is predicted to fold into a *β*-pleated sheet structure that forms a trimeric aggregate. The grooves between the subunits of the trimers serve as binding sites for the receptor [45]. Fn14 is a type I transmembrane protein. It has a single cysteine-rich domain in extracellular region (53 amino acids) that is necessary for TWEAK binding and a short cytoplasmic tail (28 amino acids) that possesses a single TNFR-associated factor- (TRAF-) binding site [2, 3].

**Figure 1:**
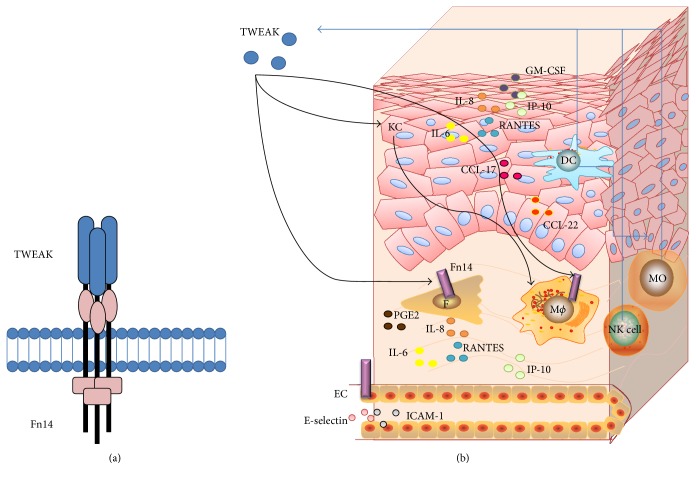
The diagram for TWEAK, Fn14, and relevant cytokines in skin structure. (a) The structures of TWEAK and Fn14 partners. (b) Fn14 is expressed on multiple cell types, including keratinocytes (KC), dermal fibroblasts (F), macrophages (M*ϕ*), and microvascular endothelial cells (EC). Intracellular TWEAK protein is expressed by monocytes (MO), dendritic cells (DC), and natural killer (NK) cells. TWEAK induces keratinocytes to express proinflammatory cytokines, such as IL-6, IL-8, RANTES, GM-CSF, IP-10, CCL17, and CCL22, which promote the migration of macrophages. TWEAK also induces the production of IL-6, IL-8, RANTES, IP-10, and PGE2 in dermal fibroblasts as well as E-selectin and ICAM-1 in microvascular endothelial cells.

The extracellular cysteine-rich domain of Fn14 contains three disulphide bonds. By analyzing the structure of Fn14, it recently revealed a highly conserved core region (Ala34–Ala69) with very few flexible side chains [46]. This region contains the residues Asp45, Lys48, and Asp62, which are particularly important for high-affinity TWEAK binding [47]. Moreover, the putative protein-protein interface in close proximity locates the side chain of Arg58, which presents a high degree of flexibility [46]. This suggests that Arg58 may act as the potential switch that opens the binding groove. Moreover, ICM-Pro algorithm (a protein structure analysis approach) was used for identifying the plausible poses of TNF ligands bound to their receptors, showing that two putative TWEAK residues, Tyr176 and Trp231, anchor TWEAK to cysteine-rich domain of Fn14 [46]. The structures of TWEAK and Fn14 molecules are diagramed in Figure 1.

## 3. TWEAK Enhances the Production of Cytokines in Keratinocytes and Other Skin Cells

Keratinocytes are the major component of the human epidermis. They secrete a broad spectrum of cytokines including proinflammatory cytokines, chemokines, and immunomodulatory cytokines and establish the local cytokine and chemokine milieu, which mediate multiple local and systemic consequences, such as migration of inflammatory cells, activation of immune responses, and proliferation and differentiation of keratinocytes and fibroblasts [48–50]. Keratinocytes can produce multiple cytokines such as IL-1, IL-6, IL-8, granulocyte-macrophage colony-stimulating factor, and transforming growth factor- (TGF-) *α* [49, 51, 52]. Keratinocytes also synthesize the C-X-C chemokines, including interferon gamma-induced protein 10 (IP-10), monocyte chemotactic protein-1 (MCP-1), and RANTES (regulated on activation, normal T cell expressed and secreted) [53]. KCs have the ability to regulate leukocyte influx in the skin by producing chemokines, such as chemokine (C-C motif) ligand (CCL) 2, CCL20, and chemokine (C-X-C motif) ligand (CXCL) 10, and this process is influenced by keratinocyte/T cell communication [54]. In addition, CCL20 activates memory T cells via its chemokine receptor 6 [55]. Therefore, keratinocytes and related cytokines are central in immunologic and inflammatory reactions in skin.

Other types of skin cells are also involved in cutaneous inflammation through secreting cytokines or other components. Dermal fibroblasts are activated under inflammatory condition and produce TNF-*α*, IL-6, and matrix metalloproteinases [56]. Fibroblasts release cytokines and growth factors that have autocrine and paracrine effects. Autocrine activity includes the TGF-*β*-induced synthesis and secretion of connective tissue growth factor which promotes collagen synthesis [57]. Paracrine activity affects growth and differentiation of keratinocytes by the secretion of keratinocyte growth factor, granulocyte-macrophage colony-stimulating factor, IL-6, fibroblast growth factor-10, and stromal cell-derived factor-1 [58, 59]. Infiltration of macrophages is a feature of skin inflammation. Macrophages produce TNF-*α*, IL-1*β*, IL-4, IL-17, IL-23, and other cytokines that may trigger or exacerbate inflammatory responses in skin [60]. Vascular injuries are commonly seen in skin diseases such as cutaneous lupus erythematosus, Henoch-Schönlein purpura, and urticarial vasculitis. These diseases are characterized by inflammatory reactions directed at small vessels, in which the damage to dermal microvascular endothelial cells is usually the primary event. Dermal microvascular endothelial cells can produce TNF-*α*, MCP-1, IL-1*α*, IL-1*β*, IL-6, and IL-8, which are fundamental in inflammation and angiogenesis [61].

Increasing evidences suggest that TWEAK significantly enhances the synthesis of cytokines in resident cells in skin tissues. TWEAK/Fn14 activation promotes the expression and/or secretion of various cytokines that are involved in inflammatory responses, including IL-6, IL-8, GM-CSF, MCP-1, and RANTES [62]. Especially, TWEAK stimulates keratinocytes to produce RANTES via Fn14 in a concentration-dependent manner and can be almost completely inhibited when blocking the TWEAK/Fn14 interaction with anti-Fn14 mAb [63]. TGF-*β*1 exhibits a synergistic effect on the TWEAK-induced RANTES production by keratinocytes [63]. TWEAK also enhances the expression of MCP-1 and IP-10 in keratinocytes [22, 64]. Moreover, TWEAK/Fn14 activation induces rapid phosphorylation of nuclear factor- (NF-) I*κ*B*α* in keratinocytes [63]. TWEAK can also induce the production of CCL2, RANTES, CCL17, and CCL20 in keratinocytes, and such effect is enhanced by synergistic signals from IL-13 and IL-17, two crucial factors in the pathogenesis of AD and psoriasis [65]. Furthermore, TWEAK deficiency ameliorates chemokine expression in skin of AD, suggesting that TWEAK functions as an upstream signal molecule [65].

The proinflammatory effects of the TWEAK/Fn14 axis have been described in other cell types. TWEAK induces secretion of prostaglandin E2 (PGE2), IL-6, IL-8, RANTES, and IP-10 in dermal fibroblasts [66]. TGF-*β* signaling increases collagen production and Fn14 expression in cultured fibroblasts, and overexpressing Fn14 can enhance the expression of extracellular matrix genes in these cells upon TWEAK stimulation [67]. Moreover, tissular macrophages express Fn14, and TWEAK can promote the production of multiple cytokines in macrophages infiltrating injured tissues [6, 68, 69], indicating that TWEAK/Fn14 activation may also affect macrophages under skin inflammation. Upon TWEAK stimulation, keratinocytes express more CCL17 and CCL22, which contribute to the local recruitment of macrophages, and further induce inflammatory responses [70]. Furthermore, dermal microvascular endothelial cells express Fn14 [71]. TWEAK upregulates expression of E-selectin and intercellular adhesion molecule-1 (ICAM-1) and even enhances the adhesion of polymorphonuclear leukocytes to microvascular endothelial cells, leading to exacerbation of skin inflammation [71]. Obviously, the TWEAK/Fn14 pathway participates in inflammatory responses through promoting the expression of cytokines or other mediators in skin cells. The expression of TWEAK and Fn14 and their interaction in skin structure are diagramed in Figure 1.

## 4. TWEAK Regulates Cell Fate of Keratinocytes via the Fn14-TRAF2-TNFR Axis

TWEAK regulates cellular responses ranging from proliferation to cell death in a manner highly dependent on the cell type and the microenvironmental context. The biological activities of TWEAK can be mediated through two structurally distinct receptor subtypes: TNFR1 and TNFR2, with the major difference in their cytoplasmic tail. Most cells of the human body express TNFR1, while the expression of TNFR2 is much more restricted [72]. Additionally, TNFR1/TNFR2 protein ratio has been found to alter under the regulation of various cytokines [73]. Both TNFR1 and TNFR2 are expressed in the synoviocytes of patients with rheumatoid arthritis, and TNF-*α* stimulation downregulates TNFR1 but upregulates TNFR2 expression [74]. These findings indicate that the TNFR1 and TNFR2 expression varies under different inflammatory microenvironments, which may generate cell fate diversity.

**Figure 2:**
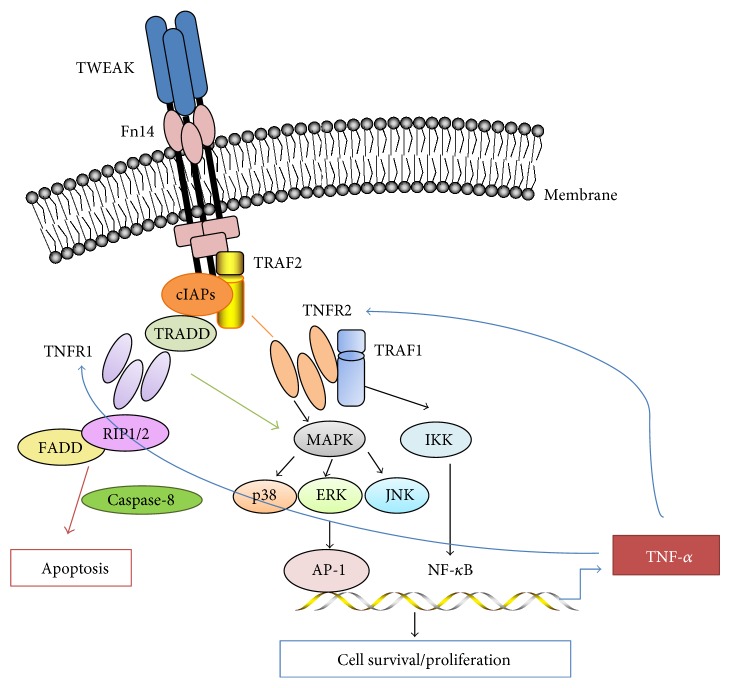
The diagram for the Fn14-TRAF-TNFR axis. sTWEAK binding to Fn14 recruits TRAF2 and cIAP1/2 to form cIAP-TRAF2 complex. The recruitment of TNFR1, TRADD, and FADD initiates apoptotic signaling by the recruitment and activation of caspase-8, while TNFR2 induces cell survival/antiapoptotic signals through NF-*κ*B activation. NF-*κ*B activation upregulates expression of multiple cellular genes that encode proinflammatory cytokines such as TNF-*α*. TWEAK may independently act or cooperate with TNF-*α* in regulating the TNFR-mediated cell fate.

The cytoplasmic domain of Fn14 contains a phylogenetically conserved binding motif, and TRAF1, TRAF2, TRAF3, and TRAF5 are able to bind to this site [3]. TRAF1 and TRAF2 are adaptor proteins that belong to the TRAF protein family. Both TRAF1 and TRAF2 are identified to be associated with the cytoplasmic domain of TNFR2 in a heterodimeric complex in which TRAF2 contacts the receptor directly, while TRAF1 interacts with TNFR2 indirectly through heterodimer formation with TRAF2 [75]. TNFR1 has a conserved motif in the cytoplasmic tail called the death domain. Upon activation, such death domain serves as a docking site for TRADD (TNFR1-associated death domain) and then recruits FADD (Fas-associated death domain protein) and caspase-8, forming the complex that initiates the cascade of apoptosis [76]. TRAF2 is an antiapoptotic protein and recruits the inhibitor of NF-*κ*B kinase complex and cellular inhibitor of apoptosis proteins (cIAP) to the TNFR1 signaling complex, thus the necessity for the activation of the classical NF-*κ*B pathway. A complex of TRAF2 with cIAP1, cIAP2, and TRAF1 has further been implicated in the inhibition of TNFR1-induced activation of caspase-8 [77]. Hence, TWEAK interacts with Fn14 in TNFR1 predominant cells that may trigger signals of apoptosis or necrosis. On the contrary, TNFR2 lacks cytoplasmic death domain. By assembling with TRAF1, TRAF2, and cIAPs, the TNFR2 signaling complex can antagonize TNFR1-induced caspase signaling and enhances cell proliferation by triggering the NF-*κ*B pathway [78, 79]. The principle of Fn14-TRAF-TNFR axis is also diagramed in Figure 2.

Recently, we found a switch of TNFR expression profile in keratinocytes under different skin inflammations. In normal keratinocytes, TNFR1 expression is prominent, leading to cell death upon TWEAK stimulation [22, 64]. Interestingly, keratinocytes prefer to express more TNFR2 but less TNFR1 under psoriatic inflammation or HPV E6/E7 transfection, which significantly promotes cell proliferation [22, 64]. These findings are in accordance with the function of Fn14-TRAF-TNFR axis.

## 5. TWEAK/Fn14 Signaling Contributes to Psoriatic Inflammation

Psoriasis is one of the most common inflammatory skin diseases. Accumulation of RANTES, IL-8, IP-10, and MCP-1 is prominent in lesional skin of patients with psoriasis [22]. Moreover, both TWEAK and Fn14 are highly expressed in these lesions [22]. TWEAK can upregulate multiple proinflammatory and chemoattractive cytokines such as CCL20 and IL-19 under psoriatic inflammation [65]. The levels of phosphorylated I*κ*B and nuclear NF-*κ*B are also elevated, indicating activation of the NF-*κ*B signaling pathway [22, 80]. NF-*κ*B activation in psoriatic inflammation results in the production of multiple proinflammatory cytokines, which further mediate the trafficking and homing of T cells, monocytes, eosinophils, natural killer cells, and mast cells [81, 82]. These findings demonstrated that TWEAK/Fn14 signals are activated in psoriatic skin lesions, and downstream proinflammatory cytokines are produced consequently.

In psoriasis, the balance between the antiapoptotic and cell cycle inhibitory roles of NF-*κ*B pathway is abnormally skewed towards the former, resulting in increased keratinocyte survival and epidermal hyperproliferation [80]. The NF-*κ*B-activated proliferation marker Ki-67 and antiapoptotic proteins (including c-Myc, survivin, cIAP-2, and cellular FADD-like IL-1*β*-converting enzyme inhibitory protein) are expressed at higher levels in keratinocytes upon TWEAK stimulation, further suggesting that TWEAK signals participate in psoriatic inflammation [22, 83]. In fact, TWEAK/Fn14 interaction promotes the proliferation to apoptosis ratio of keratinocytes under psoriatic inflammation [22].

## 6. The Function of TWEAK/Fn14 Signals in AD

AD is a chronic inflammatory disease characterized by a relapsing form of skin inflammation, disturbance of epidermal barrier function, eczema, and spongiosis formation. Histologically, AD is featured with the infiltration of T cells, macrophages, and dendritic cells. These infiltrating cells are known to secrete a range of cytokines, including IL-8, TNF-*α*, RANTES, IP-10, and interferon-*γ*, which are upregulated in the lesional skin of patients with AD [84].

Unlike other inflammatory diseases such as psoriasis or autoimmune diseases [85, 86], the circulating level of TWEAK is not elevated in patients with AD nor do they correlate with AD severity [87]. TWEAK expression has been detected not only in lesional AD skin but also in healthy skin [87]. However, Zimmermann et al. observed that TWEAK and Fn14 are highly expressed only in lesional skin [28]. Moreover, the expression of TWEAK and Fn14 increase in the skin in experimental AD, and TWEAK deficiency limits severity of AD [65]. Conflicting results might arise from different experimental procedures. This controversy needs to be clarified in further studies.

Furthermore, a significant increase of TNF-*α* mRNA in keratinocytes was observed under TWEAK stimulation, and TNF-*α* is highly expressed in lesional skin of AD but not in healthy skin [28]. Actually, excessive apoptosis in epithelium is a key feature of AD. TWEAK can cooperate with TNF-*α* in the induction of keratinocyte apoptosis, contributing to the formation of AD lesions [28]. Previous studies suggested that Fn14 does not contain a “death domain,” which directly triggers apoptosis [88]. TWEAK binds its receptor Fn14 on the cell membrane and results in an increase in the secretion of TNF-*α*, which binds to TNFR1 and triggers the extrinsic pathway of apoptosis [89, 90]. However, TWEAK also induces apoptosis or necrosis of keratinocytes without TNF-*α* [28, 89]. The highly expressed TWEAK and TNF-*α* in the lesional skin may together induce apoptosis of keratinocytes under AD inflammation. Further insight about the function of the TWEAK/Fn14 pathway in AD is expected.

## 7. TWEAK/Fn14 Pathway Mediates the Formation of Cutaneous Vasculitis

Cutaneous vasculitis includes a wide range of diseases that affect the blood vessels of skin and share a common pathological feature of endothelial damage and perivascular leukocyte infiltrates. Recent study showed that TWEAK and Fn14 are significantly expressed in the dermal vessel of lesional skin in patients with urticarial vasculitis but not in healthy controls [91]. Moreover, the serum TWEAK levels are correlated with the severity and the systemic involvement of urticarial vasculitis [91]. The similar pattern has been found in patients with cutaneous leukocytoclastic angiitis, Henoch-Schönlein purpura, and allergic vasculitis [91, 92].

Previously, it was found that TWEAK can induce the proliferation and migration of endothelial cells [93]. TWEAK treatment of human umbilical vein endothelial cells induced a rapid and intermittent increase in the expression levels of phosphorylated I*κ*B*α*, phosphorylated ERK1/2, and phosphorylated JNK1/2 and promoted cell proliferation in a dose-dependent manner; anti-human Fn14 mAb can abrogate such effect of TWEAK on human umbilical vein endothelial cells [94]. TWEAK exhibits similar effect on human dermal microvascular endothelial cell line (HMEC-1) [92]. In a human in vitro model of the blood-brain barrier, besides regulating the proliferation of endothelial cells, TWEAK/Fn14 interaction induces production of proinflammatory cytokines (CCL2 and IL-8) and is associated with an increased permeability of the monolayer formed by these cells [95]. Moreover, TWEAK/Fn14 pathway mediates the formation of cutaneous vasculitis by upregulating vascular E-selectin and intercellular adhesion molecule-1 expression in the endothelium of blood vessels [71, 95]. It has been known that upregulated adhesion molecules are instrumental factors in triggering vascular inflammation and also the key contributors in the development of cutaneous vasculitis [71]. TWEAK also enhances the adhesion of polymorphonuclear leukocytes to microvascular endothelial cells [71]. Therefore, TWEAK acts as a regulator of NF-*κ*B activation and chemokine production in human endothelial cells, thus promoting leukocyte migration and vascular injury in cutaneous vasculitis.

## 8. TWEAK/Fn14 Interaction Plays a Role in HPV Infection and Carcinogenesis

By interacting with Fn14, TWEAK is an inducer of apoptosis of keratinocytes. However, the role of the TWEAK/Fn14 pathway in regulating the cell fate of HPV-infected keratinocytes is characterized by increased proliferation instead of apoptosis [64]. Fn14 expression increases in both HPV16-positive warts and HPV16 E6/E7-harboring keratinocytes. The TWEAK levels are also elevated in HPV16-positive warts when compared with normal skin. Moreover, the downstream proteins of TWEAK/Fn14 pathway, RANTES, and NF-*κ*B are highly expressed in these areas. These findings indicate TWEAK/Fn14 activation in HPV16-infected keratinocytes [64]. Meanwhile, the level of cytoplasmic p18 (active subunit of caspase-8) is significantly reduced in E6/E7-transfected keratinocytes, suggesting the caspase-8 inhibition in TWEAK-induced cell proliferation [64]. E6 has the capacity to interact with caspase-8. In the case of HPV16, the full-length E6 protein is capable of directing caspase-8 degradation, thus preventing the apoptosis of infected cells [96]. E6 can also recruit active caspase-8 from the cytoplasm to the nuclei [97]. Based on these facts, we consider that TWEAK/Fn14 activation facilitates the proliferation of E6/E7-positive cells by inhibiting the caspase-8 pathway.

Moreover, HPV-infected keratinocytes in anogenital lesions are generally considered to be etiologically associated with carcinogenesis. The HPV16-induced keratinocyte immortalization has been suggested to correlate closely with epidermis-originated malignancies, such as cervical cancer and cutaneous or oropharyngeal squamous cell carcinoma [98, 99]. The tumor microenvironment contains certain factors that upregulate TWEAK expression, and Fn14 is overexpressed in the keratinocyte-originated cancers [100, 101]. However, in lesional skin of squamous cell carcinoma, expression of TWEAK varies significantly depending on the tumor differentiation levels. Strong staining was observed in the well-differentiated keratinocytes, while poor-differentiated tumor showed weak staining of TWEAK. It could be partially explained by the fact that the relative levels of TWEAK might induce different or even opposed cellular responses. Thus, TWEAK may also have a protective role in tumors [102]. These findings indicate that TWEAK/Fn14 interaction plays an important role in modulating cell fate in HPV infection and associated cancers.

## 9. TWEAK/Fn14 Activation Participates in Cutaneous Autoimmune Diseases

TWEAK/Fn14 activation in autoimmune diseases is strongly supported by a growing number of experimental evidences [14, 103–105]. The elevated expression of TWEAK and Fn14 in epidermis is seen in patients with cutaneous lupus erythematosus and bullous pemphigoid [8, 106]. Both TWEAK and Fn14 expressions also increase in muscles of patients with polymyositis or dermatomyositis [107]. These disorders share a similarity in inflammatory infiltration induced by chemokines that may be related to TWEAK.

Cutaneous lupus erythematosus is characterized by local activation of immune complexes or complement, autoreactive B cells and T cells and overexpression of cytokines and chemokines [108]. The TWEAK/Fn14 pathway participates in renal injuries and neuropsychiatric disease in MRL/lpr lupus-like mice [16, 38–40]. Both TWEAK and Fn14 are highly expressed in injured tissues, and their interaction induces the production of RANTES, MCP-1, and IP-10 in renal resident cells, astrocytes, endothelial cells, and other nonhematopoietic cell types [16, 38–40]. Similarly, TWEAK/Fn14 activation also exhibits effect on skin in MRL/lpr mice [14, 109]. Ultraviolet B irradiation enhances the Fn14 expression on keratinocytes in vitro and in vivo [14]. Moreover, Fn14 deficiency significantly attenuates cutaneous disease in MRL/lpr mice, as supported by the well-maintained architecture of the skin, remarkably decreased infiltration of T cells and macrophages, and less apoptotic cell in skin [14]. Furthermore, Fn14 deficiency correlates with attenuated cutaneous disease as well as reduced macrophage-derived proinflammatory chemokines (macrophage inflammatory protein-1*α*, CXCL1, and CXCL5) in skin of MRL/lpr mice irradiated with ultraviolet B [109]. These findings demonstrated that TWEAK/Fn14 signaling is important in the pathogenesis of cutaneous lupus erythematosus.

Systemic sclerosis affects skin tissue by chronic inflammation, vascular injuries, and excessive fibrosis. Recent study suggested that the interaction between sCD163 (marker of monocytes/macrophages) and TWEAK is associated with systemic sclerosis [110]. CD163 is a scavenger receptor that regulates inflammatory responses and may contribute to connective tissue remodeling. It has recently been demonstrated that CD163 can bind to and neutralize TWEAK [111]. The sCD163/sTWEAK ratio is significantly increased in patients with scleroderma, and higher sCD163/sTWEAK ratio correlates with greater skin involvement [110].

The TWEAK-Fn14 axis may be also involved in the pathogenesis of polymyositis or dermatomyositis [107]. Serum levels of TWEAK are lower in patients with polymyositis or dermatomyositis when compared with healthy controls and correlate negatively with serum CD163 levels in these patients. However, Fn14 expression increases in biopsied tissues of patients with polymyositis or dermatomyositis and correlates positively with muscle disease activity. Moreover, TWEAK protein expression is more detectable in biopsied muscle tissues of patients although its mRNA expression level does not differ from healthy controls.

Recently, we found that TWEAK/Fn14 signaling plays a pivotal role in the pathogenesis of bullous pemphigoid [8]. The serum levels of TWEAK are elevated in patients with bullous pemphigoid, and there is a positive correlation between serum TWEAK and anti-BP180 IgG. Both TWEAK and Fn14 expressions are strongly expressed in skin lesions of bullous pemphigoid. Also, TWEAK reduces BP180 expression in keratinocytes and suppresses cell adhesion, involving activation of NF-*κ*B and extracellular signal-regulated kinase pathways. Interestingly, TWEAK upregulates sheddases such as a disintegrin and metalloproteinase 17, leading to BP180 loss in keratinocytes. Therefore, TWEAK/Fn14 activation may contribute to the pathogenesis of bullous pemphigoid. The actions of TWEAK on target cell or animal models in different skin diseases are listed in Table 1.

## 10. Targeting TWEAK/Fn14 Pathway as Therapeutic Approaches

The TWEAK/Fn14 pathway has become a potential therapeutic target. Growing evidences suggest that TWEAK inhibition can ameliorate inflammatory reaction and tissue damage in several animal models of autoimmune and inflammatory diseases, such as chronic autoimmune arthritis, systemic lupus erythematosus, and experimental autoimmune encephalomyelitis [104, 105, 117]. The most important TWEAK and Fn14 targeting drug formats and their molecular mode of action include anti-TWEAK antibodies, anti-Fn14 antibodies, Fn14-Fc (a fusion protein of the ectodomain of Fn14 with the Fc domain of IgG), soluble TWEAK, and Fc-TWEAK [13]. It was found that anti-TWEAK mAb can block immune complex-induced vascular damage as well as leukocyte infiltration in murine model of cutaneous reverse passive Arthus reaction and reduces expression of proinflammatory cytokines, including TNF-*α* and IL-6, in skin lesions [118]. Anti-TWEAK mAb (BIIB023) has been applied to patients with rheumatoid arthritis in a clinical trial, and it showed a favorable safety and tolerability profile. Moreover, the downregulation of several inflammatory biomarkers (MCP-1, IP-10, MIP-1*β*, and tissue inhibitor of metalloproteinase-1) was observed in these subjects [119].

TWEAK/Fn14 signals also show therapeutic potential in the management of tumors. Firstly, agents that inhibit TWEAK binding to Fn14 may have potential therapeutic utility [120]. Anti-TWEAK antibody (RG7212) blocks TWEAK-stimulated proliferation, NF-*κ*B activation, and cytokine secretion and exhibits antitumor effect [121, 122]. Fn14-TRAIL (consists of the Fn14 extracellular domain fused to the soluble form of TNF-related apoptosis inducing ligand) also shows therapeutic potential due to its ability of inhibiting TWEAK/Fn14 signaling and promoting TRAIL signaling [123]. Furthermore, anti-Fn14 antibodies (PDL192 and BIIB036) exhibit an alternative NF-*κ*B pathway-specific agonistic activity, but do not photocopy other activities of TWEAK [124].

## 11. Conclusions and Outlook

TWEAK is a multifunctional cytokine expressed on various cell types and tissues and acts via binding to its sole receptor Fn14. TWEAK/Fn14 activation contributes to various pathological processes, including cell proliferation and death, angiogenesis, carcinogenesis, and inflammation. TWEAK/Fn14 signals are involved in the pathogenesis of multiple skin diseases including inflammatory skin diseases, autoimmune skin diseases, cutaneous vasculitis, HPV infection, and tumors. The involvement of this pathway has made it a promising therapeutic target of braking the never-ending cycle of local inflammation and tissue destruction. In a variety of the disease models, soluble TWEAK- and Fn14-specific antibodies and other drug formats have exhibited promising therapeutic effects.

However, the precise mechanism underlying the roles of TWEAK/Fn14 activation in inflammatory and autoimmune diseases, especially in cutaneous diseases, is not fully elucidated. In addition, the therapeutic effects on the inhibition or activation of TWEAK/Fn14 pathway have not been well explained. And based on the preclinical findings, we are supposed to explore the clinical value of TWEAK- or Fn14-related agents.

## Figures and Tables

**Table 1:** The action of TWEAK in different skin diseases.

Diseases	Effect on target cells or animal models	References
Psoriasis	KC: to enhance chemokine expression and cell proliferation	[22, 65]
	Murine model: to induce immune cell infiltrates in lesional skin	
AD	KC: to increase TNF-*α* expression and induce apoptosis	[28, 65]
	Dermal fibroblast: to regulate chemokine expression	
	Murine model: to induce cellular infiltrates, migration of immune cells, and chemokine expression	
Cutaneous vasculitis	HMEC: to regulate NF-*κ*B activation and chemokine production	[71, 92]
	Murine model: to induce endothelial damage and perivascular leukocyte infiltrates	
HPV infection	KC: to enhance TNFR2 expression and cell proliferation	[64]
Carcinogenesis	Various tumor cells: to induce cell proliferation or apoptosis in a cytokine-dependent way	[64, 112, 113]
	Glioma cells: to promote cell migration and invasion	
	KC: to induce cell proliferation	
	Vascular ECs: to upregulate FGF-2 and VEGF-A expression and to promote angiogenesis	
Cutaneous lupus erythematosus	KC: to enhance Ro52 and proinflammatory cytokine expression and induce apoptosis	[70, 109]
	Macrophage: to enhance chemoattraction and cytokine expression (including TWEAK)	
	MRL/lpr mice: to induce chemokine production, cell infiltration, and apoptosis	
Systemic sclerosis	Monocytes/macrophages: to lead to greater extent of skin fibrosis or to exert as a protective role against fibrosis	[111, 114]
Polymyositis & dermatomyositis	Myoblast: to induce degradation of myosin heavy chain, to affect cell proliferation and differentiation, and to induce metabolic abnormalities	[107, 115, 116]
	Murine model: to induce muscle atrophy and interstitial fibrosis	
Bullous pemphigoid	KC: to reduce BP180 expression and suppresses cell adhesion	[8]
